# Longitudinal tractography of the mouse corpus callosum reveals topographical order and differences due to sex and aging

**DOI:** 10.1007/s00429-025-03040-1

**Published:** 2025-11-04

**Authors:** Özgün Özalay, Tomas Mediavilla, Bruno Lima Giacobbo, Daniel Marcellino, Greger Orädd, Anna Rieckmann, Fahad Sultan

**Affiliations:** 1 Department of Medical and Translational Biology, Umeå University, 90 187, Umeå, Sweden; 2 Oligodendroglial Interactions Group, National Institute of Mental Health, Bethesda, MD 20892 USA; 3 Department of Nuclear Medicine and Molecular Imaging, Medical Imaging Center, University Medical Center Groningen, University of Groningen, Groningen, The Netherlands; 4 Learning and Plasticity Lab, The Department of Psychology, University of Gothenburg, 413 14 Gothenburg, Sweden; 5 Department of Diagnostics and Intervention, Umeå University, 90197 Umeå, Sweden; 6 Department of Human Sciences, University of the Bundeswehr Munich, Neubiberg, Germany

**Keywords:** Corpus callosum, White matter, Diffusion weighted imaging, Animal models, Sex

## Abstract

The corpus callosum (CC) is the major tract connecting the two hemispheres in placental mammals and diffusion-weighted (DWI) MRI has revealed the meso/macroscopic organization of human CC and its organization of connectivity with the cortex. Here, we applied longitudinal DWI probabilistic tractography to study the CC fibers of mice across the adult lifespan. Our results reveal that connections of eight cortical areas can be delineated within the CC. The mouse CC organization aligns with the human topographical organization with frontal areas occupying the genus and parieto-occipital the posterior splenium region. A further regional analysis of passage fields showed stable field sizes in many of the studied areas over time. In contrast, several areas of the mouse default mode network and motor cortical regions show a decline in size with advancing age. Our analysis also identifies sex differences in the CC with female mice showing a larger orbitofrontal commissural connection. In summary, we confirm a mammalian-like organization of the CC in mice. Furthermore, we confirm an aging-related decline in the integrity of mouse white matter that aligns with previous findings in humans, thus opening up the possibility for future developmental in vivo studies across the entire lifespan using a mouse model.

**Supplementary Information:**

The online version contains supplementary material available at 10.1007/s00429-025-03040-1.

## Introduction

The corpus callosum (CC) is the main white matter (WM) tract that bridges the two cerebral hemispheres, allowing to distribute and integrate sensory, cognitive, and motor information in the mammalian brain. The human corpus callosum contains approximately 200 million myelinated fibers and forms one of the largest long-range tracts within the brain. While long-range axons comprise only 1% of all white matter axons (Schuz and Preissl 1996; Schüz and Sultan 2009), their distant presynaptic arbors require a substantial amount of energy, which makes them particularly vulnerable to normal aging and neurodegenerative diseases, such as Alzheimer’s disease (AD) (Yang et al. 2023). Animal models of neurodegenerative diseases have been important in linking axonal/synaptic vulnerabilities to molecular and cellular pathomechanisms and metabolic origins in amyotrophic lateral sclerosis (Gurney et al. 1994) or in AD amyloidopathy (Hauptmann et al. 2009). Therefore, a precise description of the CC organization as one of the largest tracts is essential for an optimal delineation of structural damage in aging and disease in animal models.

In human work, magnetic resonance imaging (MRI) has emerged as the most suitable method to study CC structural integrity. Anatomically, the CC comprises the rostrum, the genu, the body and the splenium. The body of the corpus can be further divided into the anterior body, posterior body, and isthmus (Filippi et al. 2013). Several methods have been proposed to subdivide the CC into different geometric partitions (Clarke and Zaidel 1994; Duara et al. 1991; Rajapakse et al. 1996; Stievenart et al. 1997; Weis et al. 1993; Witelson 1989) and structural MRI has allowed expanding this analysis to in-vivo measurements. The first MRI studies confirmed the vulnerability of phylogenetically newer brain regions (Tomlinson et al. 1970), showing a sizeable age-related atrophy of the frontal lobes (Coffey et al. 1992). These findings were confirmed in the CC by the observation of a predominantly anterior reduction in CC size (Parashos et al. 1995). Subsequent studies also showed the involvement of more posterior CC regions (Biegon et al. 1994). However, early morphological analyses were not able to incorporate fiber composition or tractography of the CC which has only become possible with the advent of diffusion-weighted imaging (DWI) sequences (Basser et al. 1994a, 1994b) like Diffusion Tensor imaging (DTI). DWI tractography studies refined topological partitions of the CC by incorporating callosal parcellation with various ROI selection schemes. Notably, Chao and colleagues identified topographic subdivisions within the human corpus callosum by integrating tractography and cytoarchitectonic information (Chao et al. 2009). The streamline approach has been extended to study CC in humans in a cross-sectional study (5–59 years) using six manually delineated brain regions and showed a decline in CC diffusion parameters after a peak at 21–44 years (Lebel et al. 2010).

However, despite the transformative impact of DWI on human CC parcellation studies (and in fact WM in general), its application to longitudinal studies has remained limited. This gap likely arises from the inherent challenges of human studies, which require extensive timeframes. Imaging of animal models with short lifespans like mice (Roebroeck et al. 2025) with the imaging methods used in cross-sectional human studies provides an opportunity for understanding CC organization and changes over the entire adult period (Giacobbo et al. 2022; Özalay et al. 2024). Nevertheless, DWI longitudinal studies in mice (or other rodents) have been scarce, despite a plethora of work on modeling the WM microstructure (Arefin et al. 2023; Fukutomi et al. 2019; Mori and Zhang 2006), testing novel DWI sequences and extending DWI to connectome studies to look at the brain’s structural connectivity (Grandjean et al. 2023).

This study has two main aims: to develop a longitudinally applicable DWI protocol and analysis pipeline for the mouse brain, and to use this approach in a longitudinal setting to investigate CC organization in aging. Using this framework, we show that the mouse CC largely mirrors human topographical organization, reveals aging-related changes in white matter and exhibits sex differences in connectivity.

## Materials and methods

### Animal cohort

Sixty-six C57BL/6 J mice (46 males and 20 females) entered the study at either 6 months or 12 months. Mice were either purchased from Jackson Laboratory, California, United States (stock no: 000664, i.e., Cohort A), Charles-Rivers UK (Jax mice, stock n°: 000664, i.e., Cohort B) or bred within the Umeå Centre for Comparative Biology (UCCB). The later originated from four females and one male purchased from Charles-Rivers UK (Jax mice, stock no: 000664, i.e., Cohort C). These mice were also used for a functional MRI study (Özalay et al. 2024) and a dopamine PET/CT study (Giacobbo et al. 2022). In summary, the study combined three different cohorts: cohort A with 22 animals was scanned at 12, 18 and 24 months, while two other cohorts (Cohort B with 12 and C with 32 animals) were scanned starting at an earlier time of 6 months and followed up at 12, 18 and 24 months. The final number of data points used for DTI analyses after removing animals with failed scans due to technical difficulties or poor data quality is listed in supplementary Table 1.

Animals were housed at UCCB using standard conditions (temperature: 21 °C; 12 h/12 h dark/light cycle; water and food ad libitum). All procedures performed in this study were approved by the regional Animal Research Ethics Committee of Northern Norrland and by the Swedish Board of Agriculture (Ethical permit number: A17-2019).

### Image acquisition

Structural MRI data were acquired from animals that were either anesthetized using isoflurane (4% induction, 2% maintenance) or with medetomidine and isoflurane (0.5%). For a detailed description see Özalay et al. (2024). Animals were placed onto a cryocoil-specific MRI mouse bed (Bruker, Germany) using both tooth- and ear-bars to prevent head movement during MRI scans. The mouse was positioned inside the MR scanner (Bruker BioSpec 94/20, Germany) with the brain in the center of the field-of-view of a cryogenic RF coil (MRI CryoProbe, Bruker, Germany). We used a 220 mT/m (70 μs rise time) actively shielded gradient coil (Bruker, B-GA12S HP) of 11.4 cm inner diameter. The animal was scanned using a T1 FLASH sequence (TR/TE: 50/8 ms; flip angle: 20°; pixel dimension: 0.125 mm isotropic). DWI were acquired with a 3 segment multishot DTI_EPI sequence (TE/TR of 22.4/3000 ms and with 10 A_0_ (B = 0), 90 diffusion directions, two B-values of 1000 and 2400 s/mm^2^, 190 volumes, image matrix of 96 × 48, 83 coronal slices with an isotropic voxel size of 0.18 mm) and a total acquisition time of 28 min and 30 s.

After the scan, the animals were administered 0.3 mL of 1:20 atipamezole (Atipam, Dechra Veterinary Products, Sweden) diluted in 0.9% saline to induce awakening and rehydrate the animal. The animal was placed into a recovery cage until fully awake, and then returned to its home cage.

### DWI preprocessing

DICOM images were exported in paravision and then converted to nifti files (MRIcron’s dcm2niix). The 10 B0 volumes were averaged to create a no-diffusion image (nodif). The nodif images of all scans (6, 12, 18 and 24 months old) were used for template head (skull and brain) creation for optimal registration and subsequent skull extraction. This study-specific nodif template was created with the antsMultivariate-TemplateConstruction script (Advanced Normalization Tools (ANTs) version >  = 2.1 1 (Avants et al. 2011)) using the following parameters: 3 dimensional registration, four iterations and initial rigid body registration. Following the creation of the head template, a brain mask was manually segmented on the template image and then registered to the individual subject spaces using linear and non-linear deformation matrices derived from the template creation process (Avants and Gee 2004). Brain masks were checked visually and were manually adjusted if required. In a second template creation step we used masked images to create a brain template. The registration matrices derived from this step were subsequently used for all other templates for individual registrations.

Further DWI preprocessing steps included drift signal correction with linear detrending (Vos et al. 2017), denoising with dwidenoise (MRtrix3; (Veraart et al. 2016)), Gibbs ringing removal with mrdegibbs and dwifslpreproc (MRtrix3; (Tournier et al. 2019)) with eddy current correction (Andersson and Sotiropoulos 2016). Final outcome images were manually checked for artifacts (such as missing brain regions).

### Probabilistic tractography

The GPU version of bedpostx (FSL, vs. 5.0, (Jenkinson et al. 2012)) tool was used to estimate distributions on diffusion parameters at each voxel (Hernández et al. 2013) and local probability density functions with the diffusion tensor model (Behrens et al. 2007). To perform probabilistic tractography, the GPU version of the probtrackx tool was used on the bedpostx outputs. Probtrackx provides streamline probability matrices with the number of tracts traced between the seed and target regions. The curvature threshold was set to 0.2/90degree, the number of seed repetitions to 5000 and the number of steps to 2000 (Behrens et al. 2007). The step length was adjusted to the voxel dimensions (0.09 mm), and fibers were prevented from looping back.

### Population fiber densities

Segmenting the CC to derive the parcellations started with a coarse mask (template CC_co_) that was manually delineated on the nodif template (see Supplementary Fig. 1). The border was placed loosely to include surrounding tissue to compensate for subsequent back-registration deformations (nodif template to individual DWI images), which could lead to losing CC tissue. A co-registered coarse mask (individual CC_co_) was then used as the seed region for tract tracing. To derive target tracts, we used 36 neocortical areas (18 for each hemisphere) from the Allen atlas (Lein et al. 2007; Özalay et al. 2024) that were registered to template space and then to the subject individual space (using linear and non-linear deformation matrices derived from the brain template creation process). CC tracts were traced to left and right targets separately. A mid-sagittal exclusion ROI (sparing the CC coarse mask and registered to the individual space) was applied to prevent any tracking outside of the CC.

To obtain the average CC streamlines, we registered the number of streamlines per CC voxel and individual back to the template space by applying linear transformation matrices derived from template creation. We then obtained the population-based probabilistic tractography for each side by summing and normalizing the probabilities and dividing them by the number of individuals. This approach follows previous probabilistic tractography studies of the corpus callosum (Chao et al. 2009; Park et al. 2008). The probabilistic tractography was calculated by normalizing the number of streamlines (p) for each CC voxel (v_i_) connecting to Allen atlas brain regions b_j_ by the total amount of streamlines p found between v_i_ and all atlas regions:

1 $$P\left({v}_{i},bj, s\right)=\frac{p\left({v}_{i},{b}_{j},s\right)}{\sum_{j=1}^{b}p\left({v}_{i},{b}_{j},s\right)}$$

The probabilistic tractography P was thresholded (thr_m_) at multiple thresholds (0.1 till 0.2 in steps of 0.025) to minimize a single threshold bias:

2 $$\overline{P }\left({v}_{i},{b}_{j}, s,{thr}_{m}\right)=\left\{\begin{array}{c}1, P\left({v}_{i},bj, s\right) > {thr}_{m} \\ 0, P\left({v}_{i},bj, s\right) < {thr}_{m},\end{array}\right.$$

The population-based probabilistic tractography (Ṗ) is estimated by taking the average over all thresholds thr_m_ and all individual (s):

3 $$\dot{P}\left({v}_{i},bj\right)=\frac{1}{S}\frac{1}{M}\sum_{i=1}^{S}\sum_{m=1}^{M}\overline{P }\left({v}_{i},{b}_{j}, s,{thr}_{m}\right).$$

### Corpus callosum parcellation

The CC parcellations were derived by combining left and right sides. This was done by taking the voxelwise product of the population-based probabilistic tractography Ṗ for each side and by then applying a threshold of 0.05^2. In a second step we used Matlab’s *bwboundaries* (with the ‘noholes’ option) to obtain regional boundaries.

### Statistics

We tested for the influence of sex and age on streamline densities and CC field size using multi-level regression with linear mixed-effects (LME) models. Mixed-effects analyses are particularly suitable for longitudinal studies with repeated measurements and unequal follow-up schedules (Aarts et al. 2014; Gueorguieva and Krystal 2004). As described above, animals were enrolled into the protocol either at 6 or 12 months of age and underwent a variable number of scans (1–4), due to both dropout (death or technical difficulties) and the addition of new cohorts in order to retain sufficient numbers into very old ages. This resulted in an unbalanced design, with differences in baseline age and longitudinal depth across animals. LME regression accommodates missing data, unequal group sizes, and irregular follow-up intervals by modeling both fixed effects (sex, age at scan, or both) and random effects (subject-specific intercepts). Importantly, because age at scan directly reflects the time elapsed since baseline, the age effect in the model captures the longitudinal trajectory of each animal across the lifespan. This approach allowed us to leverage all available data while controlling for inter-individual variability. Models were implemented in Matlab using *fitlme* with maximum likelihood estimation. A significance threshold of α = 0.05 was applied, with false discovery rate (FDR) adjustment for multiple comparisons (Benjamini and Hochberg 1995).

## Results

Sixty-six mice (46 males and 20 females) entered the study for repeated scanning at either 6 or 12 months. A total of 152 DWI scans were collected (see Supplementary Table 1): 31 DWI scans were successfully obtained from mice at six months of age, 57 DWI scans at 12 months, 35 DWI scans at 18 months and 29 at 24 months, respectively. On average the mice were scanned 2.3 times with 20 mice scanned either two or three times each and nine mice were successfully scanned four times. Sixteen mice could only be scanned once and were nevertheless included to improve base-line assessment. Across all scans, the average age was 14.5 months.

**Fig. 1:**
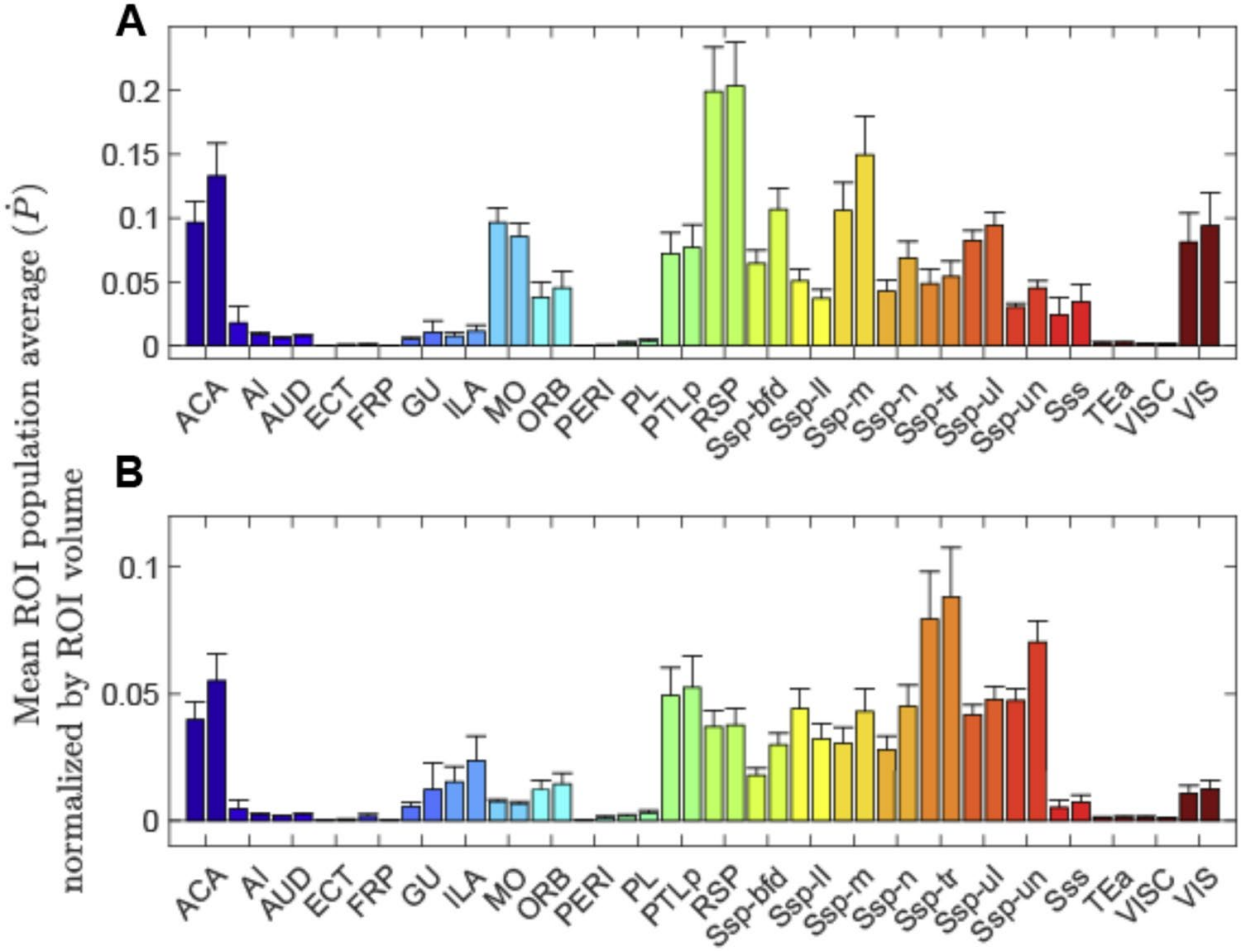
CC ROI averages of population-based probabilistic tractography. **A** shows the mean of all voxels of the population-based probabilistic tractography of the 48 studied ROIs. Each cortical area is shown with the left and right sides depicted as separate bars (left and right bars of the same color). **B**: mean of all voxels normalized by the size (number of voxels) within a ROI. The primary somatosensory area (SSp) is plotted with its seven separate somatotopic ROIs. The analysis shows that about 13 cortical areas (and subregions) had detectable streamlines passing through the CC (choosing a mean above 0.01 as the threshold). Abbreviations: ACA (Anterior cingulate area); AI (Agranular insular area); AUD (Auditory areas); FRP (Frontal pole, cerebral cortex); GU (Gustatory areas); ILA (Infralimbic area); MO (Somatomotor areas); ORB (Orbital area); PL (Prelimbic area); PTLp (Posterior parietal association areas); RSP (Retrosplenial area); SSp-n (Primary somatosensory area, nose); SSp-bfd (Primary somatosensory area, barrel field); SSp-ll (Primary somatosensory area, lower limb); SSp-m (Primary somatosensory area, mouth); SSp-ul (Primary somatosensory area, upper limb); SSp-tr (Primary somatosensory area, trunk); SSp-un (Primary somatosensory area, unassigned); Sss (Supplemental somatosensory area); TEa (Temporal association areas); VISC (Visceral area) and VIS (Visual areas). Error bars show the SEM

The population-based probabilistic tractography (Ṗ) showed a largely similar tractography density for the left and right cortical areas (Fig. 1). Of 48 studied cortical areas, 13 showed detectable streamlines through the CC (with Ṗ > 0.15). For eight of these areas (ACA, ILA, MO, ORB, PTLp, RSP, SSp, VIS), a voxelwise analysis within the CC identified specific clusters within CC through which the streamlines pass (see below); these are denoted as CC “passage fields” from hereon. The other five (AI, AUD, GU, PL, SSs) areas show few streamlines that appear widespread throughout CC (Supplementary Fig. 2, Ṗ < 0.08 at any one voxel). They are not analyzed further.

### Streamline population results (Figs. 2 and 3)

**Fig. 2:**
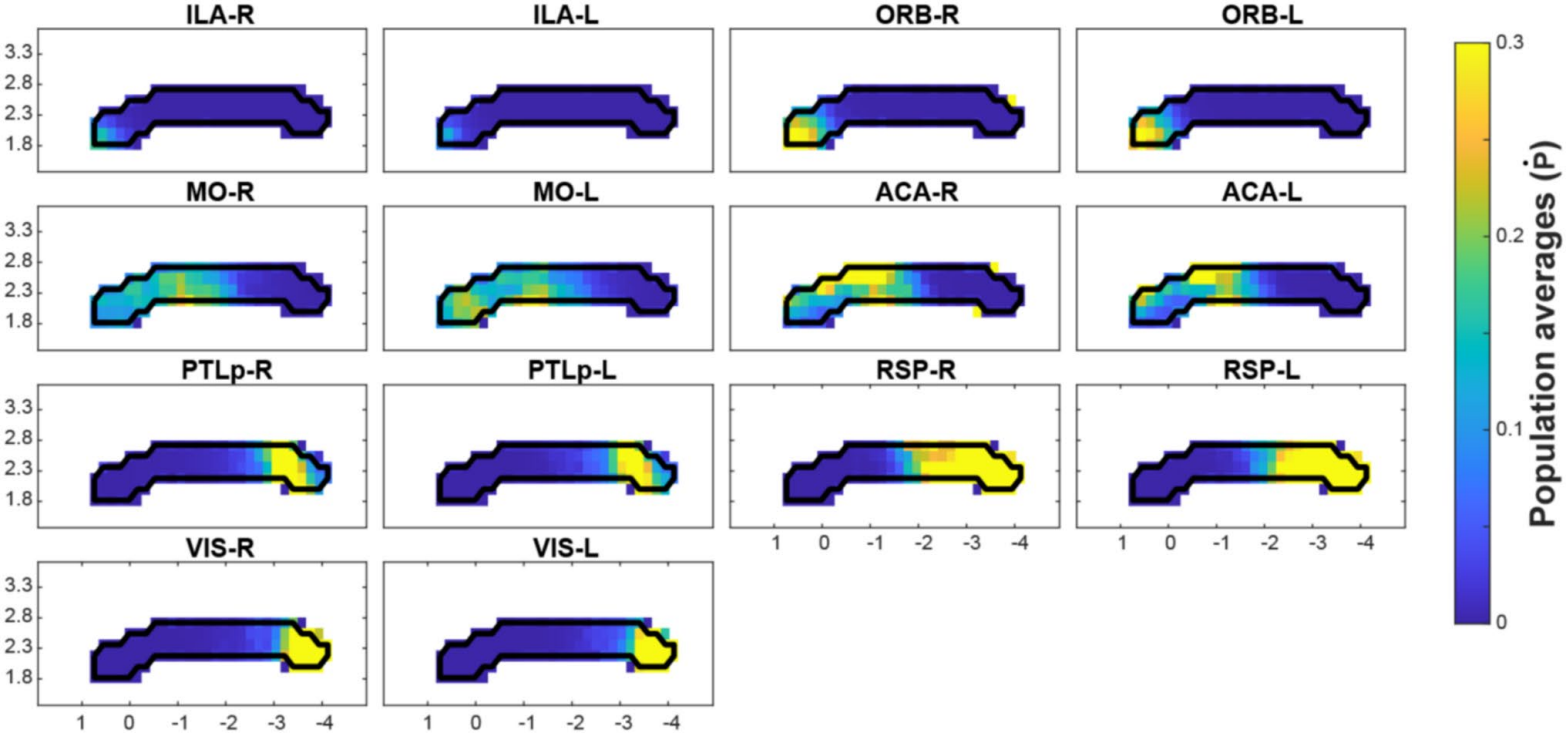
Population-based probabilistic tractography of CC. CC connections of seven cortical areas are shown from areas with detectable projections (without primary somatosensory areas) ordered according to the location of the termination region (anterior to posterior). CC projections show different patterns, with some areas having limited regions within the CC (ILA, ORB, PTLp and VIS), while others show a larger spread (MO, ACA, RSP). In general, these patterns are reproducible when comparing their left and right hemispheric origin. Data plotted are from 152 MR scans and from 66 individuals. Axis denotes location relative to the anterior commissure (placed at [0 0] origin)

**Fig. 3:**
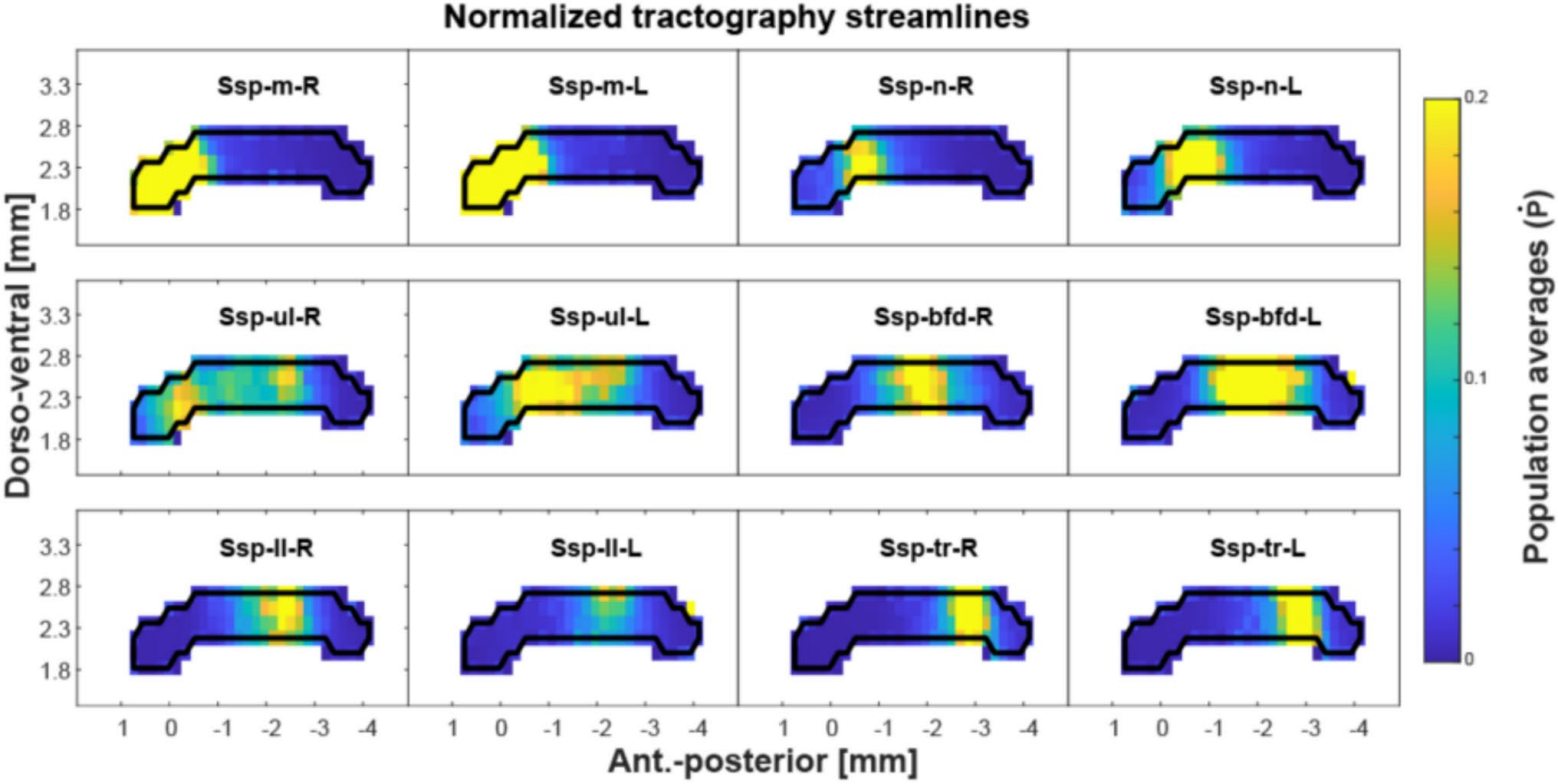
Population-based probabilistic tractography reveals somatotopically organized primary somatosensory CC connections. SSp area mouth (m), nose (n), upper limb (ul), barrel field (bfd), lower limb (ll), and trunk (tr) are organized in an anterior–posterior fashion covering most of the CC except for the posterior splenium. Note that most regions show very similar patterns when comparing left and right hemispheric projections. The upper limb and barrel field part show some deviation from this symmetry. Axis denotes location relative to the anterior commissure (placed at [0 0] origin)

The identified CC passage fields are plotted in Figs. 2 and 3. The cortical areas are ordered based on their passage field’s location (Fig. 2) from anterior to posterior. Figure 3 shows different SSp body parts also ordered by their passage fields’ location. The SSp passage fields confirm an orderly projection of mouth, nose, upper limb and barrel field regions located anteriorly and lower limb and trunk posteriorly.

### Symmetry of corpus callosum passage fields (Figs. 4)

**Fig. 4:**
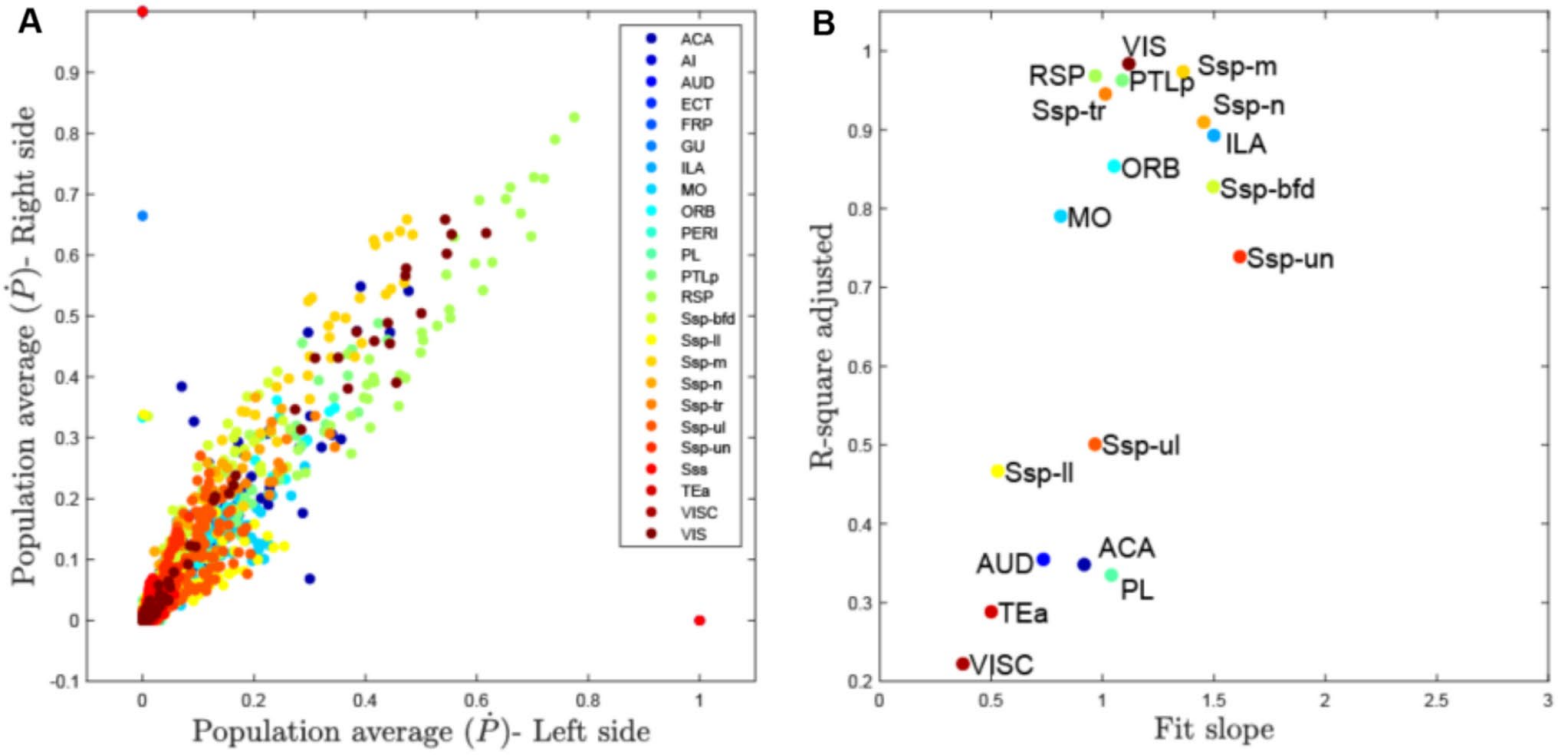
Bilateral symmetry of CC connectivity by cortical area. In A the population average for each CC voxel and each area of the left and right sides are plotted. In B we plotted the results of the linear fits to the left–right population averages. Several areas show a fit with a slope close to 1, indicating the CC voxel receives a similar number of streamlines from the respective left and right cortical areaa. Ten areas/subregions are within a slope of 1 ± 0.5 (ORB, ILA, MO, RSP, SSp-m, SSp-n, SSp-bfd, SSp-tr, PTLp and VIS) and with an R^2^ above 0.7

Figures 2 and 3 suggest largely consistent results for left and right hemispheric CC passage fields’ location and intensity distribution. The symmetry was quantified by correlating Ṗ of the left and right sides (Fig. 4). Figure 4a shows the voxel wise left and right Ṗ values, while Fig. 4b shows the fit results of the linear regression through left–right Ṗ voxels for each area. The fit slope and goodness of fit (R-squared adjusted) are shown, with a slope of 1 indicating symmetry with equal Ṗ coming from the left and right sides. Our results identify a cluster of areas with high symmetry at r2 > 0.7 (ILA, RSP, ORB, PTLp, VIS and s subregions of the SSp (mouth, nose and trunk)).

### Corpus callosum parcellation and age-related changes (Figs. 5, 6)

**Fig. 5:**
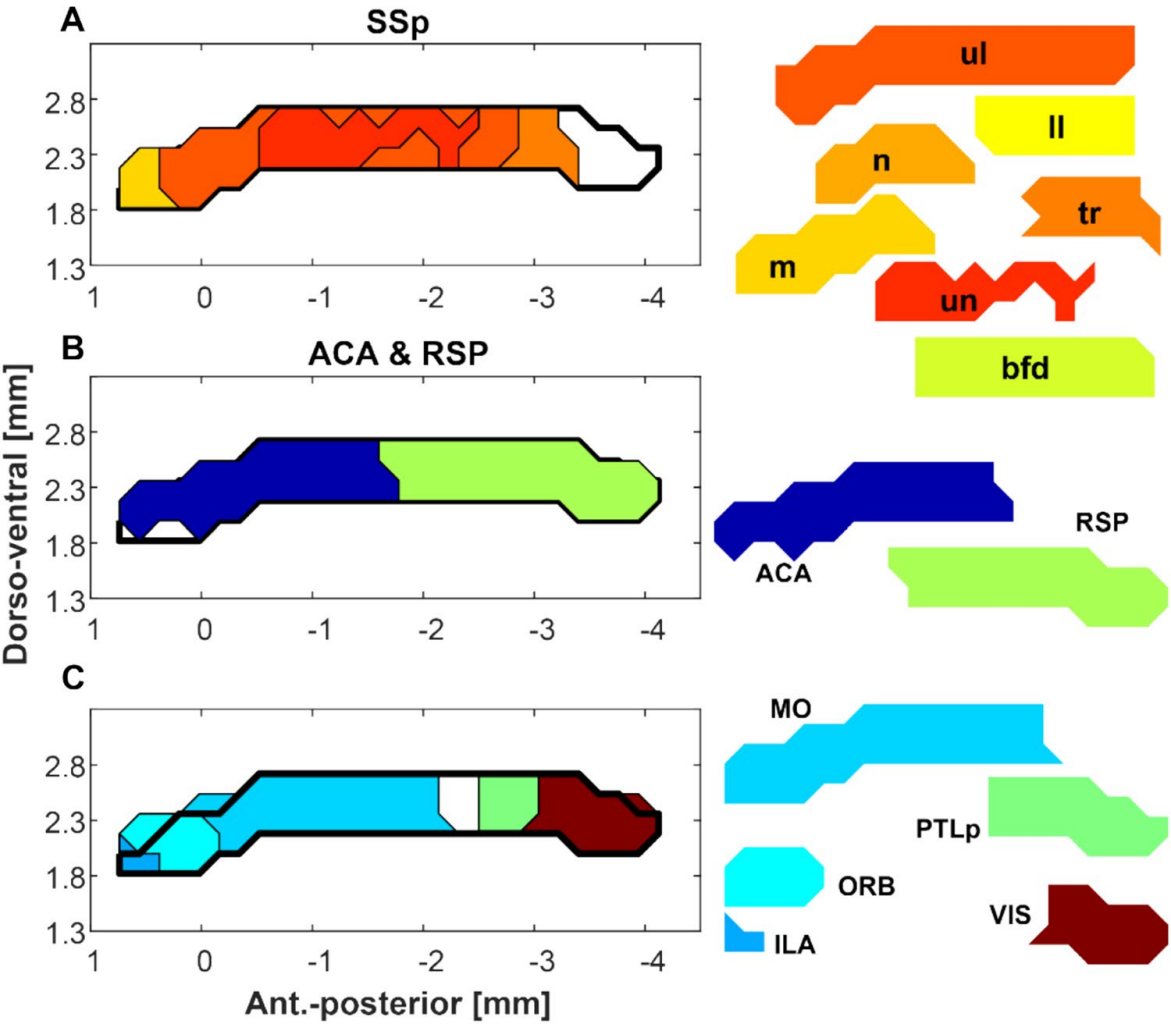
Parcellation of cortical CC passage fields. Population averages were obtained by combining left and right tract densities (by taking voxel by voxel product and thresholding them at 0.05^2). Thresholded regions are shown in three separate groups: primary SS (**A**), ACA and RSP (**B**) and ILA, ORB, MO, PTLp and VIS areas in **C**. Passage fields (right panels) are displaced (dorso-ventrally) for better identification. The axis in **A**–**C** denotes the location relative to the anterior commissure (placed at [0 0] origin). **D** The number of voxels within each parcellation was quantified and their development during aging is shown. Several parcellations show a stable size as animals grow older (SSp_ll, SSp_ul, ILA, PL, PTLp and VIS), while others either showed a decline (ACA and MO) or an increase (SSp_n, SSp_bfd) with age. **E** Parcellation size was further modeled with a linear-mixed effects test and the effects of age, sex and their interaction were studied. Our data revealed no sex and age interaction. Plotted are the t-values of the linear-mixed effects test with those values in BOLD that showed significance (p < 0.05) after FDR correction. ORB fields were larger in females than in males and ACA, RSP and MO showed a significant decline with age (p < 0.05, FDR corrected). The field size increase with age in SSp_n and SSp_bfd was also significant (p < 0.05, FDR corrected)

**Fig. 6:**
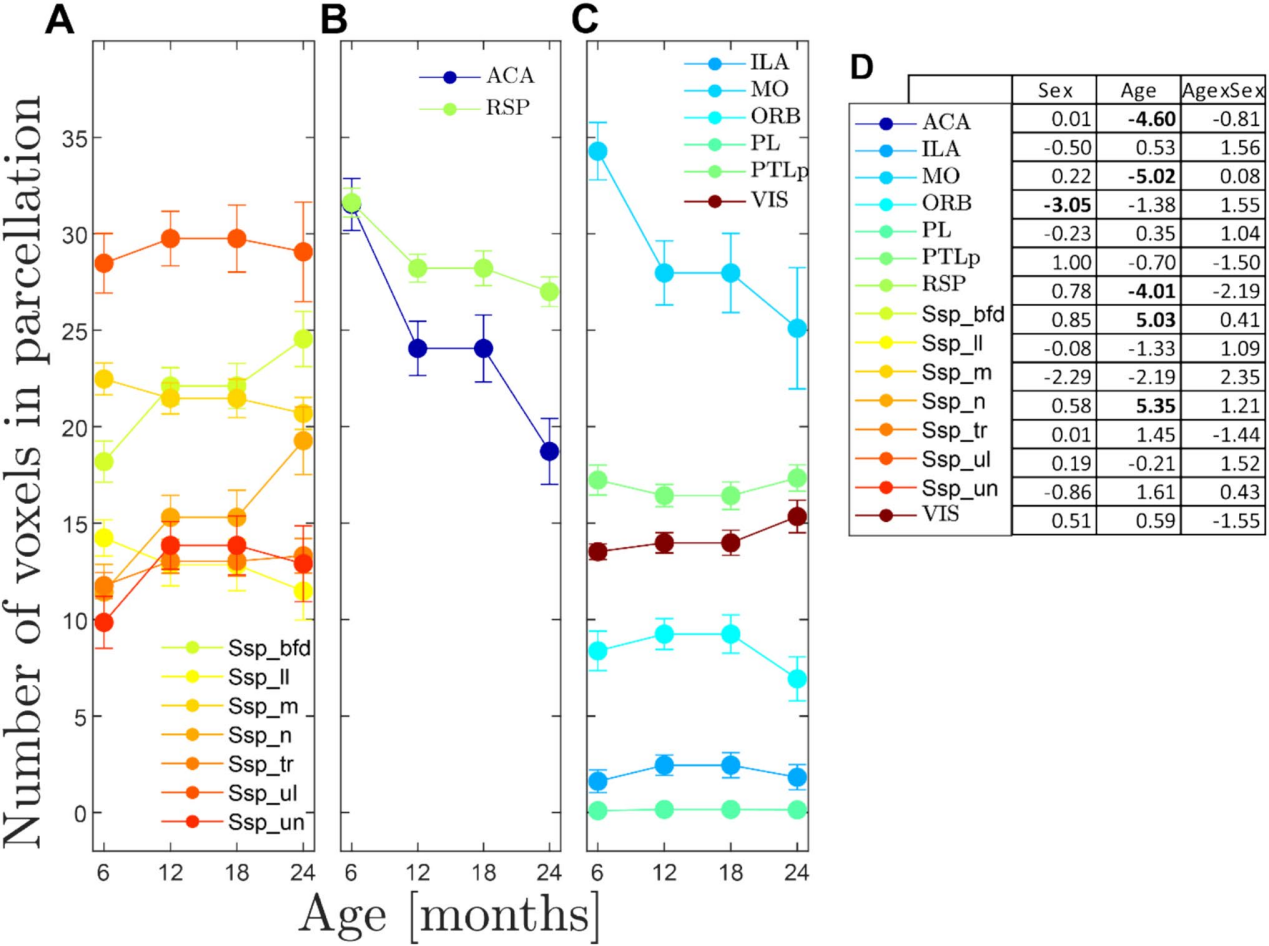
Quantification of parcellation of cortical CC passage fields. **A**–**C** The number of voxels within each parcellation was quantified and their development during aging is shown. Several parcellations show a stable size as animals grow older (SSp_ll, SSp_ul, ILA, PL, PTLp and VIS), while others either showed a decline (ACA and MO) or an increase (SSp_n, SSp_bfd) with age. **D** Parcellation size was further modeled with a linear-mixed effects test and the effects of age, sex and their interaction were studied. Our data revealed no sex and age interaction. Plotted are the t-values of the linear-mixed effects test with those values in BOLD that showed significance (p < 0.05) after FDR correction. ORB fields were larger in females than in males and ACA, RSP and MO showed a significant decline with age (p < 0.05, FDR corrected). The field size increase with age in SSp_n and SSp_bfd was also significant (p < 0.05, FDR corrected). Error bars show the SEM

Maps of the CC passage fields were obtained by thresholding the population-based probabilistic tractography densities and deriving the boundaries. The densities of the left and right sides were combined by taking the voxel-wise product and thresholding at 0.05^2. Figure 5a–c shows the maps separated into different groups. The SSp (Fig. 5a) has a large field that fills out the majority of the anterior 3/4 of the CC. ACA and RSP (Fig. 5b) have passage fields that comprise separate halves of the CC. Similarly, the MO (Fig. 5c) also has a large field within the CC that overlaps with ACA and SSp. In contrast separate fields are occupied by the infralimbic and orbital areas in the anterior region and the visual and posterior parietal cortex in the posterior region of the CC (Fig. 5c).

The age-related changes in the passage fields were quantified and the results are shown in Fig. 6a–c. Figure 6 plots the parcellation field size (number of voxels within a parcellation) depending on age. Stability, increases or decreases were identified by applying LME regression with field size as outcome and age and/or sex as fixed effects. The regression outcomes (t-values) are listed in the table in Fig. 6d and results with significant outcomes (p < 0.05, FDR corrected) are shown in bold font. In general, the regressions showed no significant interaction between age and sex. In contrast, age had different effects on field size. We observed significant negative effects on ACA, MO, and RSP, while positive associations (i.e., increases) between age and field size were found in primary somatosensory areas SSp-Bfd and SSp-n. Sex had a significant main effect on the ORB field size with males having a smaller size than females (t = -3.05; p < 0.05, FDR corrected).

### Effects of sex and age on CC streamline densities (Figs. 7, 8)

**Fig. 7:**
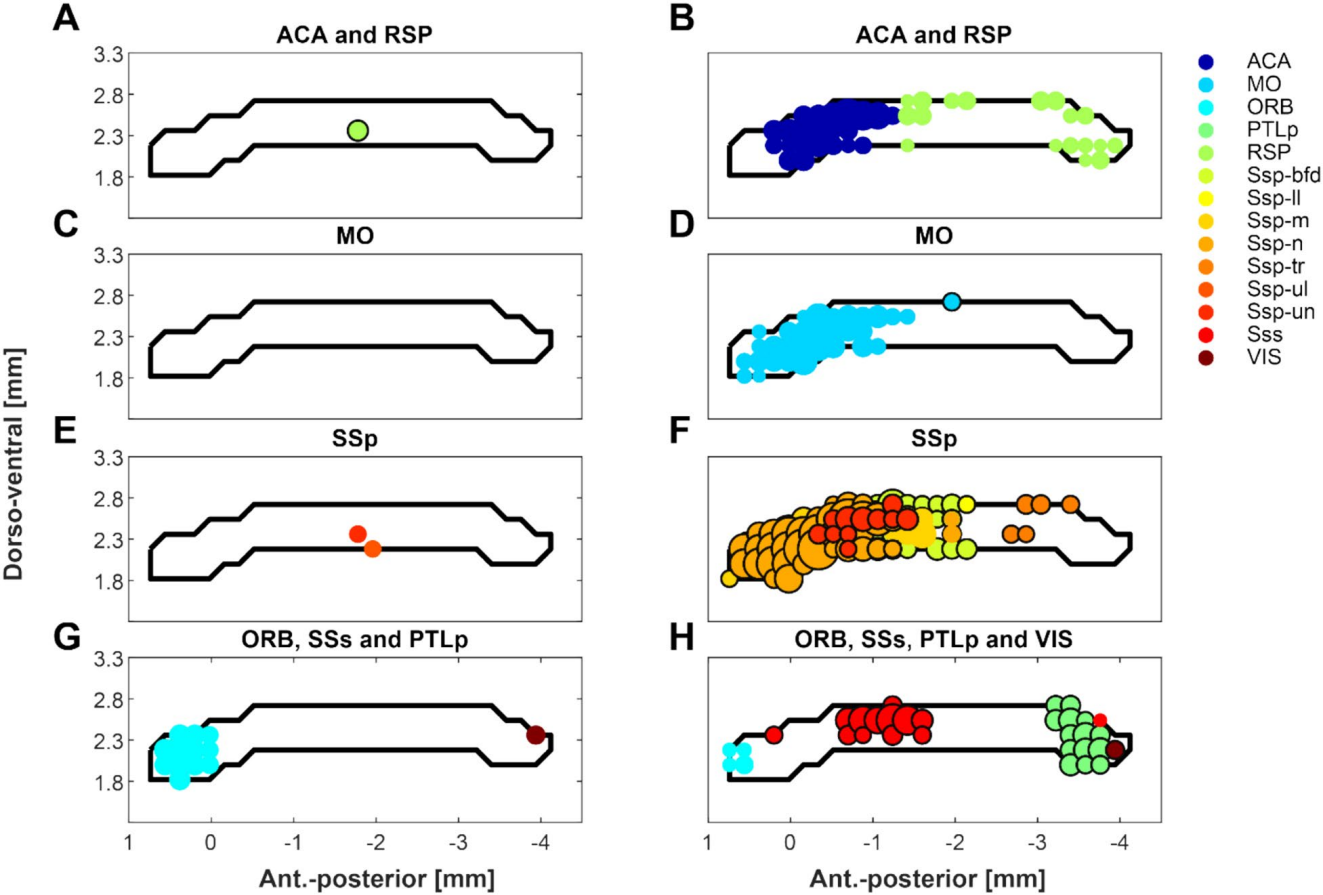
Sex and age comparison of CC densities by cortical areas. **A**–**H** plots the voxels of a cortical area that showed a significant linear mixed effect of sex or age (p < 0.05, FDR corrected) on their average tract densities. Voxel-wise comparison of sex (left column panels: **A**, **C**, **E**, **G**) and age (right column panels: B,D,F,G) of tract densities within the CC separated into four groups. A, C, E, G: sex differences were largely seen in ORB with higher densities in females. **B**, **D**, **F**, **H**: Age effects were largely seen as density decreases in ACA, RSP, MO and ORB. Increases were seen in several SSp subregions (SSp-n, SSp-bfd, SSp-m, SSP-tr) and in SS and PTLp. Increases are plotted with circles with a black border and decrease as circles without border. In the sex analysis males were taken as the reference level (male increases are shown with a black border)

**Fig. 8:**
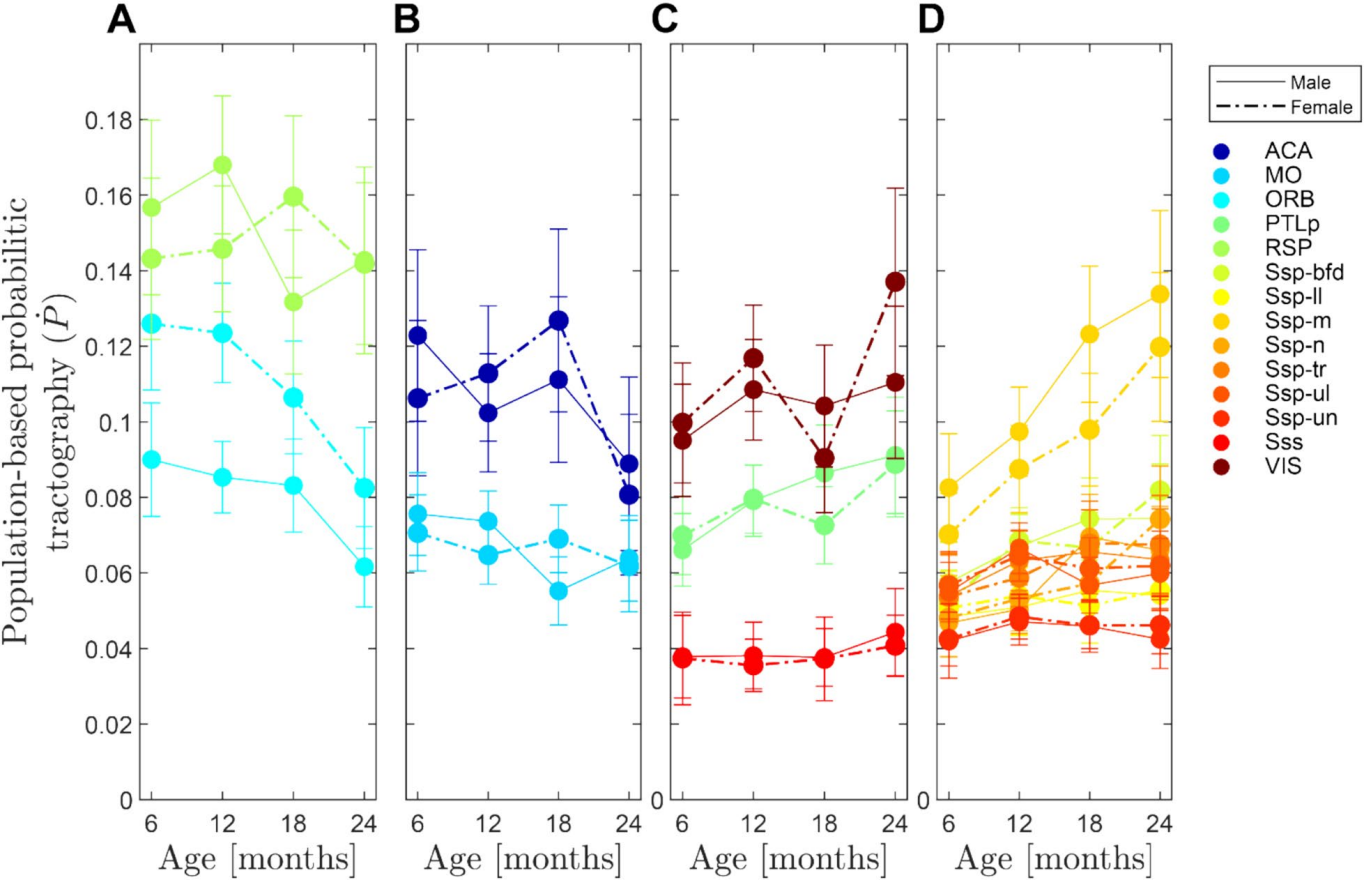
Quantification of CC densities by cortical areas. Plots A-D show the average densities within each passage field separated into three groups for better distinction. A: This confirms the decline in ACA and ORB and shows higher ORB densities among females (dash-dotted line) than males. B, C: shows a decline in MO, while PTLp and Sss show an increase in densities with age. Plot D also confirms the increase for several SSp areas (SSp-m, SSp-n and SSp-bfd). Error bars show the SEM

In addition to the associations of age and sex with passage field size explored above, we analyzed streamline densities of the CC fields (Fig. 7a, c, e, g). A linear-mixed effects regression with sex or age as fixed effects on the different target areas of the CC confirmed our CC size analysis and showed a larger density of tracts in the female ORB field (Fig. 7g). We tested the effect of sex on ORB streamline densities by moving a male group to the females in a similar fashion as done previously to explore a confounding effect of housing (Özalay et al. 2024). The new grouping confirmed that sex still exhibited a stronger fixed effect than the new grouping with 12 ORB voxels showing a significant sex effects with a mean t-value of = -4.16, in contrast to a reduced seven voxels that showed significance with the new mixed grouping (with a t-value of -3.9).

Age effects were observed in several ROI passage fields (Fig. 7b, d, f, h) and included age-related decreases and increases in population-based probabilistic tractography densities. In line with decreases in field size, we observed decreases in tract density in ACA, RSP (Fig. 7b), and MO (Fig. 7d). In addition, tract density decreases with advancing age were observed in ORB (Fig. 7h) despite statistically stable field size (Fig. 5D-E). Increases in density were observed in primary somatosensory areas. Compared with the field size analysis, the density analysis also identifies changes in SSp-m, Sss and PTLp (Fig. 7f, h), suggesting that streamline density is a more sensitive marker of CC aging than field size alone. Plotting the averages for each ROI’s population-based probabilistic tractography density (Fig. 8a-d) confirmed the decreases with age in ORB, ACA and MO (Fig. 8a, b) and the increases for PTLp, Sss (Fig. 8c), and SSp subregions (Fig. 8d). Summary of the statistics is listed in Table 1. Excluding the mice that were scanned only once yielded very similar tractography densities (see Supplementary Fig. 3).

**Table 1:**
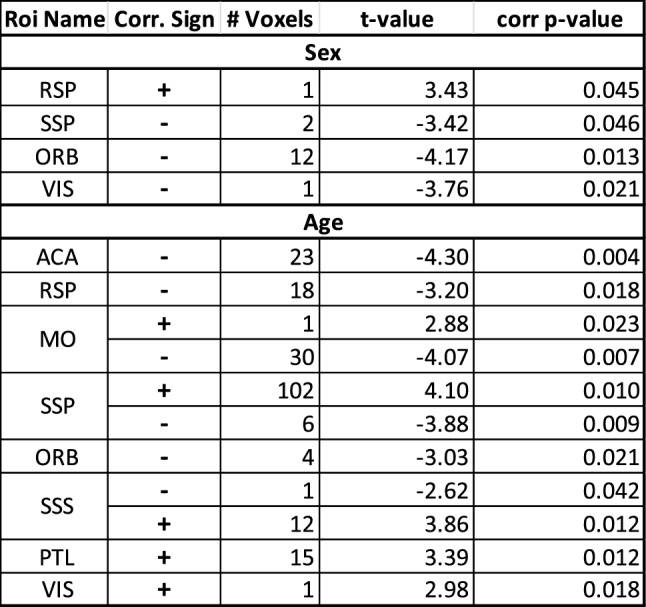
Summary statistics of CC densities by cortical areas

## Discussion

This study examined the topography of the mouse CC and identified sex- and age-related changes using in vivo longitudinal MRI across the adult lifespan. Our findings demonstrate that the organization and topographic structure of the mouse CC align with previously described features in placental mammals (Barakovic et al. 2021; Innocenti et al. 2022; Matsunami et al. 1994). Specifically, we observed an anterior–posterior organization of several CC projection areas, including the ACA, ILA, MO, ORB, PTLp, RSP and VIS. Additionally, we identified a similar topographic arrangement within subregions of the primary somatosensory area. Our analysis further revealed significant sex- and age-related changes in CC field size and streamline density. These findings are particularly relevant as they highlight the potential of the mouse model for longitudinal studies investigating white matter changes across the lifespan and their implications for behaviour and disease.

The structural organization of the CC—comprising the genu, corpus, and splenium—is a conserved feature across mammals, including both rodents and primates. We find that in the mouse, as in primates (Caminiti et al. 2009), the genu and splenium contain projections from slightly more cortical areas (4–5) than the midsection (3). Specifically, the genu transmits fibers from frontal regions including ILA and ORB, as well as MO, SSp, and ACA, while the corpus connects SSp, MO, ACA, and RSP. The splenium carries projections from RSP, PTLp, and VIS. This general organization suggests that the CC maintains a degree of topographic consistency across species, with functionally distinct cortical regions sending projections through specific subregions of the CC. The relative prominence of the genu and splenium connections in the mouse CC could also suggest that frontal and parietal cortical regions played an evolutionary significant role in rodent behavior. However, this interpretation assumes that cortical areas are well-delineated, which is not always the case. For example, the VIS region in the Allen atlas likely comprises multiple distinct visual areas which may result in thinner projections due to functional heterogeneity within the selected ROI (Glickfeld and Olsen 2017; Wang et al. 2011).

Despite these structural similarities across species, a key comparative question is whether mammals with larger brains, such as primates, exhibit greater segregation of cortical area passage fields or whether they show a similar degree of overlap as observed in the mouse. This question has clinical significance, particularly in determining whether discrete CC fields exist in humans that contain fibers originating exclusively from a single cortical area, allowing for more specific functional analyses not possible in a translational approach. Caminiti and Innocenti (Caminiti et al. 2013; Caminiti et al. 2009) mapped CC projections in monkeys using tracer injections and compared them with DWI. Their findings revealed largely segregated passage fields, with prefrontal projections clearly separated from motor and premotor areas, while some overlap was observed between parietal and temporal regions as well as among different visual areas. These findings suggest that primates exhibit a more specialized interhemispheric connectivity pattern compared to mice, potentially due to differences in brain size, functional specialization, and evolutionary pressures. In contrast, the smaller mouse brain may inherently show greater overlap in CC passage fields, reflecting less compartmentalization of cortical projections.

In addition to similarities and differences in organization, sex differences in CC connectivity provide further insights into functional specialization. We observed a stronger ORB projection in females, suggesting that frontal regions may play a different role depending on sex. The observation that this cortical region is important in shaping sociability in female mice (Jeon et al. 2023) may be in line with our observed enhanced CC connectivity in females and their distinct functional network aging pattern (Özalay et al. 2024).

We can confirm an age-related decline in streamline densities (and passage field size) in tracts coming from areas of the mouse DMN: ACA, RSP and ORB; (Grandjean et al. 2020; Mandino et al. 2022)); as well as the MO. In humans, the DMN includes medial prefrontal, posterior cingulate and bilateral areas of the temporal and inferior parietal regions and is activated at rest and deactivated during attention demanding tasks (Brown et al. 2015; Buckner et al. 2008; Menon 2023; Raichle et al. 2001). Our findings of longitudinal changes within the mouse DMN connections across the lifespan are in line with cross-sectional human evidence (Abellaneda-Pérez et al. 2019; Beason-Held 2011; Ereira et al. 2024). DMN function is also altered in multiple neurodegenerative and psychiatric diseases (Abellaneda-Pérez et al. 2019; Brown et al. 2015; Chen et al. 2019; Giorgio et al. 2024; Greicius et al. 2004; Jones et al. 2011; Kanno et al. 2021; Tang et al. 2021). Furthermore, the DMN has been shown to be present in most mammals studied so far with some variations (Lu et al. 2012), including the addition of the ACA in the rodent DMN (Grandjean et al. 2020; Özalay et al. 2024). The human DMN vulnerability has been attributed to the required long range axons connecting prefrontal, parieto-occipital and temporal regions (Yang et al. 2023). This, however, may not be the case in the rodent brain where the DMN areas are situated closer to each other and additional mechanisms are likely present.

Our analysis also identified an increase in population-based probabilistic tractography/streamline densities of several cortical areas up to 18 months of age, with some regions (SSp) continuing their increases to 24 months. This finding shows that DWI-MRI can detect life-long white matter changes and confirms previous results in mice of cortical myelin plasticity up into old age (Hill et al. 2018). Human aging studies of the CC also showed an increase in myelination into old age with a subsequent late onset of structural decline beginning in the 7-8th decade (Bloch and Friedrich 2024; Laissy et al. 1993; Parashos et al. 1995; Salat et al. 1997; Weis et al. 1993). Moreover, functional MRI studies in humans have identified a relative sparing of primary sensory networks in comparison to association networks like the DMN (Chan et al. 2014; Pedersen et al. 2021).

Our study used tractography rather than estimates of CC size or DTI microstructural parameters (such as FA) to explore the effects of age and sex. The population-based probabilistic tractography approach allows the disentangling of CC regions containing a mixture of fibers from different cortical regions, i.e., solves the problem of overlap in passage regions (e.g., of MO and SSp with frontal cortical areas) and thereby allows the detection of changes in specific areas. Our study shows that this is also possible in regions of the CC where both increases and decreases in white matter co-occur. Previous studies of WM and aging have focused on microstructural parameters (e.g., FA, MD) analysis (Lebel et al. 2010; Rieckmann et al. 2016). The mouse brain would allow us to combine DTI imaging with ex-vivo histological analysis to better understand how fiber increases and decreases during aging relate to these parameters and future studies will be required. Finally, the analysis of age effects revealed subtle changes in streamline density not captured by field size alone. Future research should explore the additional sensitivity of streamline density also in relation to CC shape (Davatzikos et al. 1996; Demeter et al. 1988) or the relative size of CC subregions (Aukland et al. 2011; Weis et al. 1993).

The resolution available for DWI tractography imposes certain limitations. For instance, a mesoscopic resolution of DWI will bias towards larger and myelinated axons (Azadbakht et al. 2015). The abundance of symmetric connections in our projection patterns may indicate a preference for this method for homotopic CC connections. This may be due to the fact that such axons run in joint tracts or alternatively they may have larger diameter axons. Currently we have not analyzed our data to search for heterotopic projections and therefore cannot exclude their tractability. Studies in humans and monkeys were able to track such fibers with DWI (De Benedictis et al. 2016; Innocenti et al. 2016) promising the expandability of this method to also cover heterotopic projections.

A further limitation of streamline-DWI analysis of CC connections is its sensitivity. Our study only detected a minor projection from areas such as the auditory cortex, which is known to have commissural projections (Rüttgers et al. 1990). Potentially this also occurred in other cortical areas with weaker connections that were not detected with this method (Azadbakht et al. 2015) and would require validation with tracer studies. However, alternate DWI protocols (e.g., with more or higher b-values) may help to alleviate this specific problem. An additional caveat exists due to the spill-over (kissing) of nearby tracts. This is pronounced in the small mouse brain where some major tracts such as the fornix and the hippocampal commissure run close by the CC. These tracts touch upon the ventral side of the mouse CC (Davatzikos et al. 1996). This artifact may be prevented by including exclusion masks or, as in our case, limiting the targets to neocortical areas. A similar problem arises from our atlas-based approach with some regions being a composition of regions with different CC connectivity properties. This would be the case for visual areas (VIS) and also the barrel field regions of the SSp, with the latter being a composition of an acallosal center and callosal peripheral regions (Schüz et al.^2006^; Yorke and Caviness 1975).

## Supplementary Information

Below is the link to the electronic supplementary material.

Supplementary file1 (DOCX 421 KB)

## Data Availability

Exemplary MRI raw data are available via the OSF (Sultan 2024) data sharing service or upon reasonable request. The scripts employed for DWI data analysis and codes used to generate figures and statistical analysis are available upon reasonable request.

## Abbreviations

ACA: Anterior cingulate area
AI: Agranular insular area
AUD: Auditory areas
FRP: Frontal pole, cerebral cortex
GU: Gustatory areas
ILA: Infralimbic area
MO: Somatomotor areas
ORB: Orbital area
PL: Prelimbic area
PTLp: Posterior parietal association areas
RSP: Retrosplenial area
SSp-n: Primary somatosensory area, nose
SSp-bfd: Primary somatosensory area, barrel field
SSp-ll: Primary somatosensory area, lower limb
SSp-m: Primary somatosensory area, mouth
SSp-ul: Primary somatosensory area, upper limb
SSp-tr: Primary somatosensory area, trunk
SSp-un: Primary somatosensory area, unassigned
Sss: Supplemental somatosensory area
TEa: Temporal association areas
VISC: Visceral area
VIS: Visual areas
