# Supplementary material for: Longitudinal tractography of the mouse corpus callosum reveals topographical order and differences due to sex and aging
Source: Brain Struct Funct. 2025 Nov 4;230(8):170. doi: 10.1007/s00429-025-03040-1 (PMC12586224; doi:10.1007/s00429-025-03040-1)
Supplement: Supplementary file 1 — Supplementary file1 (DOCX 421 KB) [file 429_2025_3040_MOESM1_ESM.docx]

## Supplementary material

### Supplementary Figure 1: Placement of CC mask

###
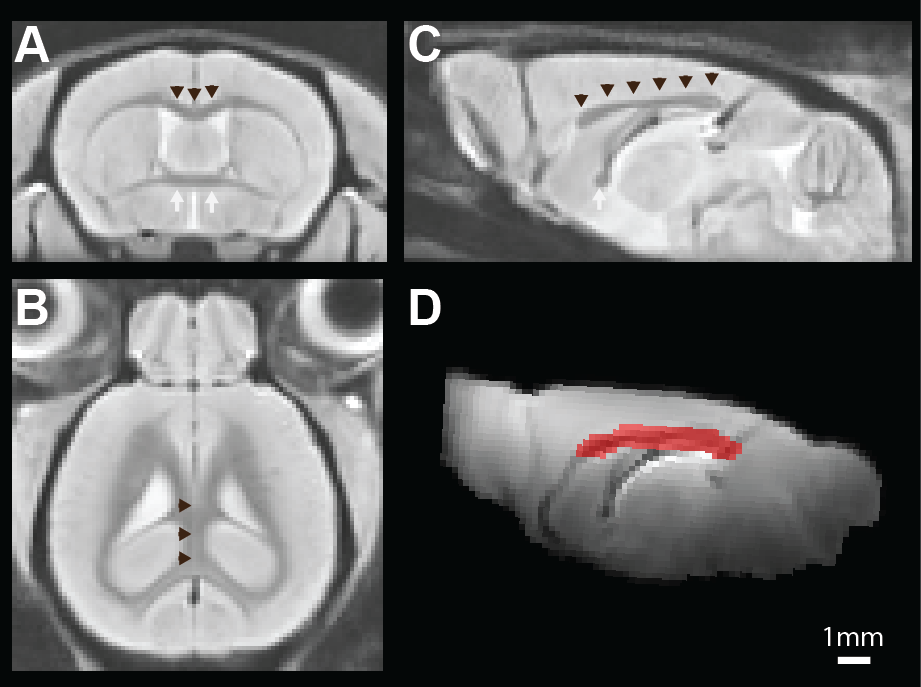


**A-C**: Shows the T1 study-specific template with coronal (A), axial/horizontal (B), and sagittal (C) views. Black arrowheads delineate CC, and white arrows the anterior commissure. The CC mask was manually delineated on the nodif template (D) with a loosely placed border to include surrounding tissue (red ROI in D). The area delineated on the nodif template was 4mm^2^ compared to the CC area on the T1 template with 2mm^2^.

### Supplementary table 1: Summary of DTI scans per mice source and cohort numbers per age group

| **Cohort (sex)** | **Source** | **6 months** | **12 months** | **18 months** | **24 months** |
| --- | --- | --- | --- | --- | --- |
| **A (M)** | S1 |  | 17 | 15 | 6 |
| **B (F)** | S2 | 10 | 11 | 6 | 6 |
| **C (F)** | S3 |  | 8 | 4 | 7 |
| **C (M)** | S3 | 21 | 21 | 10 | 10 |
| **Total** |  | 31 | 57 | 35 | 29 |

**S1**: Jackson Laboratory (stock n°: 000664, California, U.S.A.); **S2**: Charles-Rivers (stock n°: 000664, Kent, UK); **S3**: Bred at UCCB (Charles-Rivers; stock n°: 000664, Kent, UK).

UCCB: Umeå Centre for Comparative Biology; Unclassified into either cohort were an additional 6 mice.

### Supplementary Figure 2:


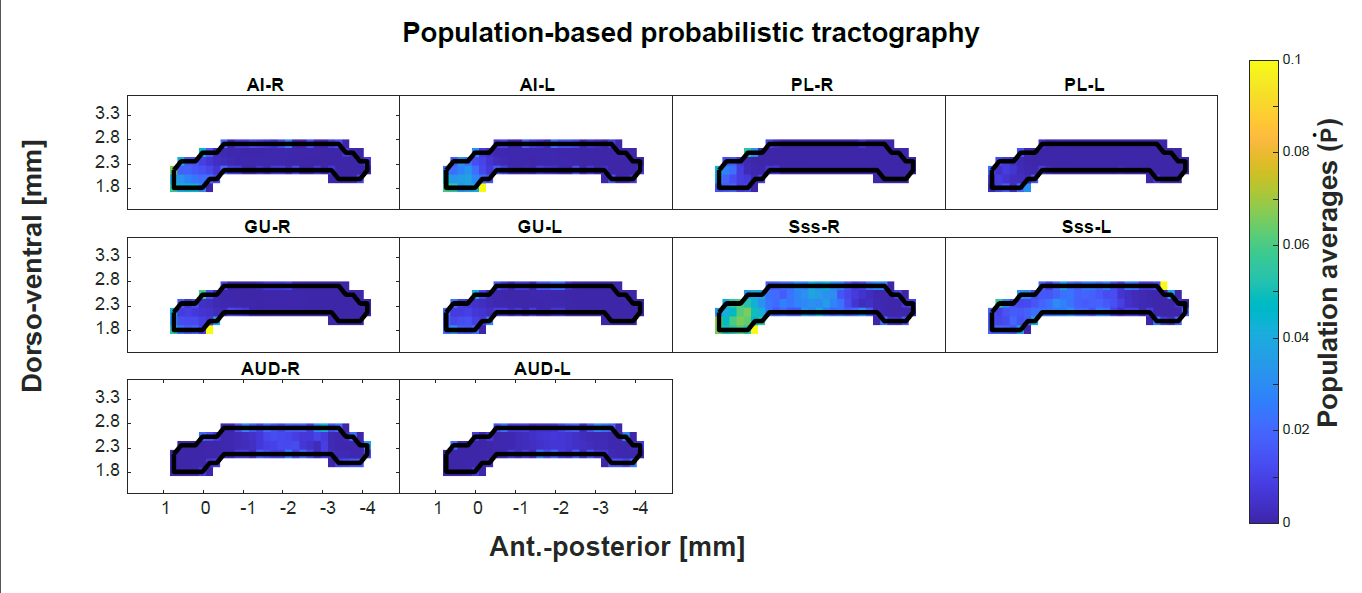


Plot shows population based probabilistic tractography of five ROIs with weaker CC tracts (ROIs not included in Figs 2 and 3).

### Supplementary Figure 3:


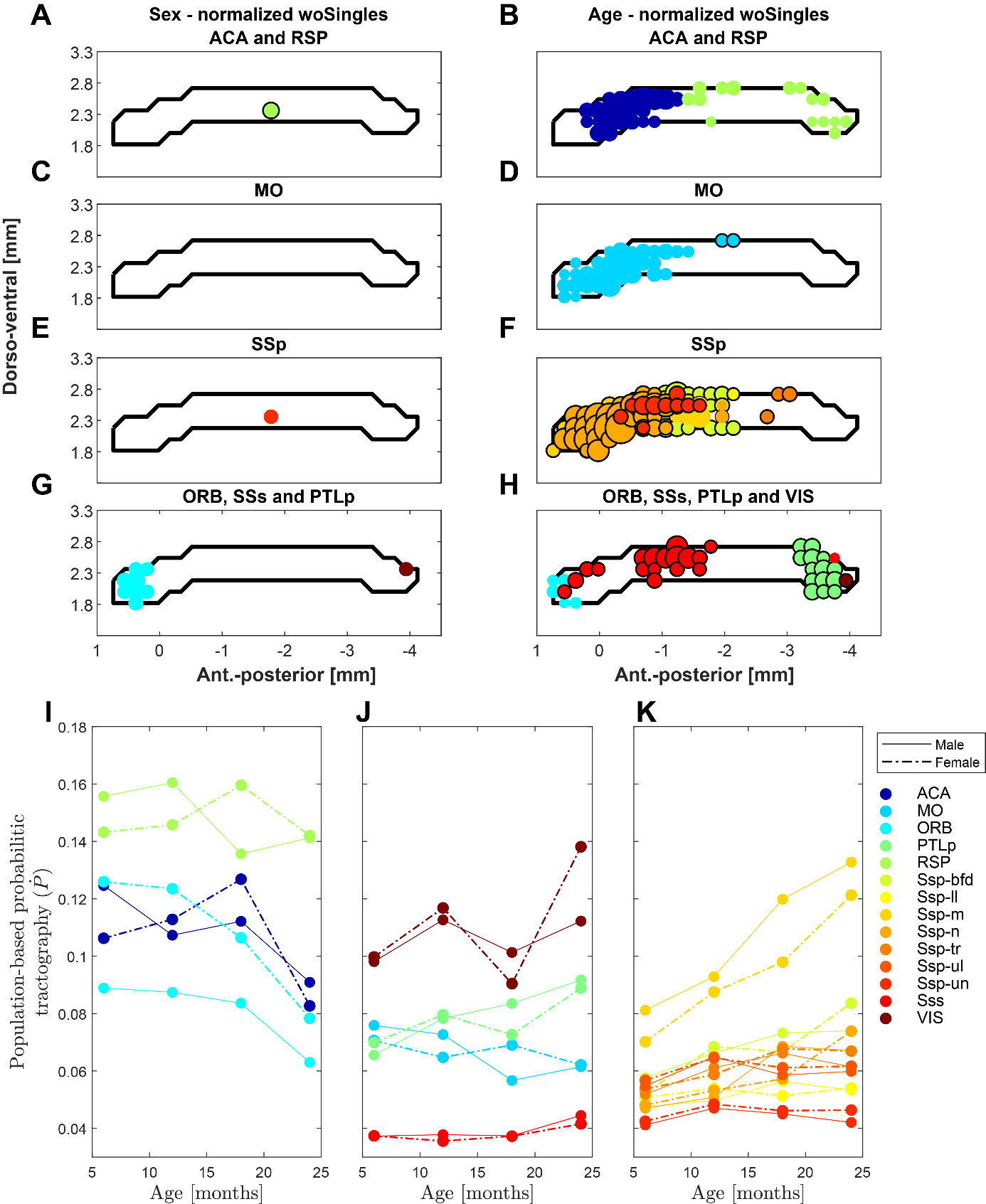


Sex and age comparison of CC densities by cortical areas without individuals that had only single time-point scans. Data here includes 136 DWI scans from 50 individuals. For further details refer to figure legend 7 and 8.
